# PRL3 as a therapeutic target for novel cancer immunotherapy in multiple cancer types

**DOI:** 10.7150/thno.79265

**Published:** 2023-03-21

**Authors:** Pei Ling Chia, Koon Hwee Ang, Min Thura, Qi Zeng

**Affiliations:** Institute of Molecular and Cell Biology, Agency for Science, Technology and Research (A*STAR), Singapore 138673; chiapl@imcb.a-star.edu.sg; angkh@imcb.a-star.edu.sg; mthura@imcb.a-star.edu.sg

**Keywords:** Phosphatase of Regenerating Liver-3 (PRL3), cancer metastasis, monoclonal antibody (mAb), immunotherapy, clinical trials

## Abstract

Phosphatase of Regenerating Liver-3 (PRL3) was discovered in 1998 and was subsequently found to be correlated with cancer progression and metastasis in 2001. Extensive research in the past two decades has produced significant findings on PRL3-mediated cancer signaling and functions, as well as its clinical relevance in diverse types of cancer. PRL3 has been established to play a role in many cancer-related functions, including but not limited to metastasis, proliferation, and angiogenesis. Importantly, the tumor-specific expression of PRL3 protein in multiple cancer types has made it an attractive therapeutic target. Much effort has been made in developing PRL3-targeted therapy with small chemical inhibitors against intracellular PRL3, and notably, the development of PRL3-zumab as a novel cancer immunotherapy against PRL3. In this review, we summarize the current understanding of the role of PRL3 in cancer-related cellular functions, its prognostic value, as well as perspectives on PRL3 as a target for unconventional immunotherapy in the clinic with PRL3-zumab.

## Background

PRL3, encoded by Protein Tyrosine Phosphatase 4A3 (PTP4A3), is a member of the class of dual-specificity protein tyrosine phosphatases known as the phosphatases of the regenerating liver (PRLs). These phosphatases facilitate reversible dephosphorylation of proteins, a key post-translational modification that regulates multiple cellular processes, including but not limited to transcription, cell cycle progression, and important signaling pathways 1. PRL3 was identified by Zeng *et al* in 1998 2. It is composed of 173 amino acids with a molecular mass of 19,535 Da. PRL3 is prenylated *in vivo* and* in vitro*, a post-translational modification that enables the association of PRL3 with the cell membrane 2, 3. The active site of PRL3 contains a catalytic cysteine residue at position 104 that is important for phosphatase activity, and a single amino acid substitution of this cysteine for serine could render PRL3 inactive, thereby perturbing its cellular functions 4, 5. Importantly, PRL3 was first linked to cancer progression and metastasis by Vogelstein in 2001 6. Since then, numerous studies have firmly established PRL3 as an important oncoprotein. In the subsequent sections, we will discuss the tumor-specific expression of PRL3 in various cancer types, its central role in regulating cellular functions and signaling pathways in cancer, as well as therapeutic targeting of PRL3 via unconventional antibody-based immunotherapy.

## Tumor specific expression of PRL3 protein in multiple cancer types

To date, there has been extensive research done on PRL3 regarding its expression in primary and metastatic tumors, prognostic value, and function in cancer cells. High PRL3 expression has been associated with both primary and secondary tumors of approximately twenty different cancer types, a higher risk of metastatic outcome, and poor prognosis.

The Zeng group analyzed PRL3 protein expression in multiple human tumor tissues (n = 1008) by immunohistochemistry (IHC) of formalin fixed paraffin embedded samples and detected an average of 22.3% PRL3 overexpression 7. Many other labs have also assessed PRL3 protein expression in tumor tissue sections mainly using IHC and found a highly variable positivity rate of 16-90% **(Table 1)**. By western blotting (WB) of fresh-frozen tumor samples, it was found that PRL3 protein was consistently highly expressed (80.6%) across 11 common cancer types but not in patient-matched normal tissues 8. This suggests PRL3 is a cancer specific therapeutic oncotarget. The same highly specific antibody against PRL3 9 yielded a differential positivity rate when used in WB (80.6%) and IHC (22.3%) analyses. This indicates a potential high chance of false negatives (~58%) with IHC detection of PRL3 **(Table 2)**. Such a discrepancy was attributed in part to non-representative tumor sections and failed antigen retrieval on formalin-fixed paraffin-embedded (FFPE) sections. Because formalin-fixed tissue undergoes tissue processing before embedment in paraffin (wax), antigen cross-linking, and epitope masking could occur. Variable protocols between laboratories and/or individuals as well as protocols non-optimized for different tissue types are additional confounding factors 10. Furthermore, the use of different commercially available antibodies can impact results as some antibodies have low sensitivity and/or cross-react with other PRLs. Therefore, potential false negatives in IHC assessment suggests the percentage of tumors expressing PRL3 protein is at the higher end of the range derived from numerous studies. It also implies that a full WB to detect the 20 kDa PRL3 protein band could provide a more accurate assessment of PRL3 protein expression in clinical diagnosis. Importantly, studies have consistently found minimal or no PRL3 protein expression in patient-matched normal tissues, which affirms PRL3 as a highly tumor-specific target and suggest targeting PRL3 is a promising strategy for effective treatment of many cancer types with potentially few side effects.

## Cellular functions regulated by PRL3

**Figure 1:**
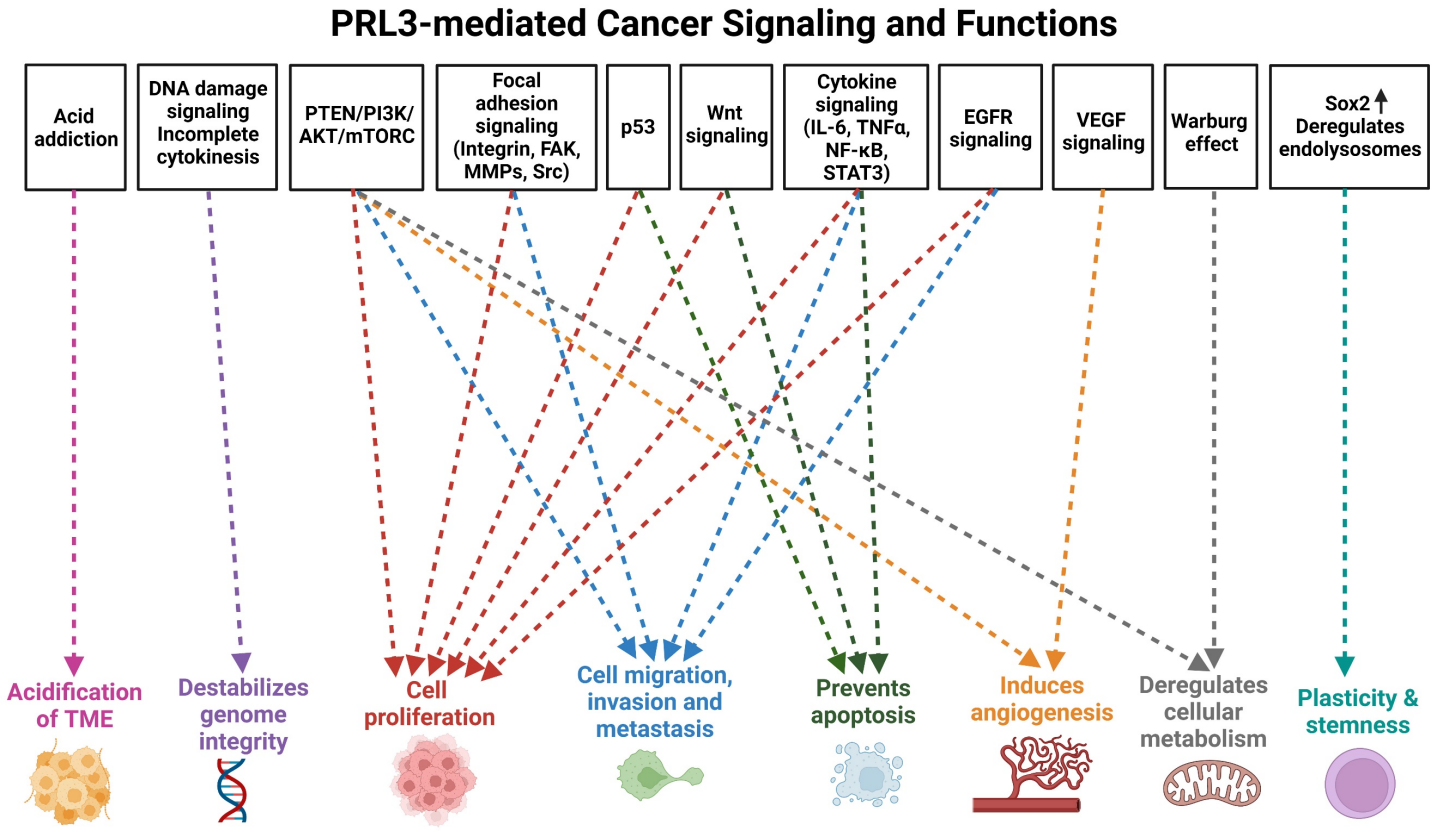
** PRL3-mediated cancer signaling and functions.** Overview of cellular functions perturbed by PRL3 in cancer progression. Created with BioRender.com

In addition to the assessment of clinical samples, studies utilizing genetic and chemical perturbation methods in both *in vitro* and *in vivo* models have established a causative role for PRL3 in promoting cancer progression. PRL3 overexpression perturbs many cellular functions that are deregulated in cancers, leading to phenotypes such as increased cell proliferation, migration, invasion, metastasis, cell cycle deregulation, evasion of apoptosis, genomic instability, angiogenesis, deregulation of cellular metabolism, phenotypic plasticity, as well as alterations in the tumor microenvironment that are permissive for tumor growth and metastasis **(Figure 1)**. These features are widely considered to be key hallmarks of cancer 35-37, thereby attesting to the role of PRL3 as an oncogenic hub regulating a web of downstream processes that facilitate cancer progression and metastasis. In the sections below, we provide an update from earlier publications and reviews that have discussed the cellular functions and signaling pathways disrupted by PRL3 overexpression 38, 39.

### Cell proliferation and tumorigenesis

In 2001, Matter *et al* reported a role for PRL3 in promoting cell proliferation; HEK293 cells transfected to overexpress PRL3 exhibited increased growth rate, and this was dependent on PRL3 phosphatase activity 40. Since then, several papers have further demonstrated that PRL3 promotes proliferation and tumorigenicity *in vitro* and *in vivo* in various cancer cell models, including mouse melanoma cells 41, 42, Chinese Hamster Ovary (CHO) cells, and human cancer cell lines including liver 14, ovarian 20, 43-45, gastric 46, 47, lung 24, 48, colon 49-53, breast 54, prostate 31, 55, esophageal 22, multiple myeloma (MM) 56, 57, glioma 58, classical Hodgkin lymphoma 34, Acute Myeloid Leukemia (AML) 59-61, Chronic Myeloid Leukemia (CML) 62, and T cell acute lymphoblastic leukemia (T-ALL) 63.

### Cell migration, invasion and metastasis

The causative role of PRL3 in mediating cell migration, invasion, and cancer metastasis is well established. The Zeng group reported this phenomenon in 2003; stable expression of PRL3 in CHO cells increased cell migration and invasion *in vitro*, and promoted tumor formation and cancer metastasis *in vivo* 4. Subsequently, these observations were confirmed in other cancer cell models both *in vitro* and *in vivo*, including liver 14, gastric 17, 46, 47, 64-66, prostate 31, 55, colorectal 11, 51, 53, 67-72, esophageal 22, lung 24, diffuse glioma 58, breast 54, 73, melanoma 41, 74-76, ovarian 77, nasopharyngeal 78, B-cell Acute lymphoblastic leukemia (B-ALL) 32, AML 79, MM 23, and classical Hodgkin lymphoma 34.

In parallel, a clinical association between PRL3 and cancer metastasis has also been established. This association was first made by the Vogelstein lab, who found that PRL3 mRNA was expressed in metastatic colorectal cancer (CRC) but not in benign tumors or normal colorectal epithelium in 2001 6. Their findings were corroborated by various studies involving human samples, including hepatocellular carcinoma (HCC) 13-15, intrahepatic cholangiocarcinoma 16, CRC 11, 51, gastric cancer 64, 80, prostate cancer 31, esophageal squamous cell carcinoma 22, non-small cell lung cancer 24, and cervical cancer 25.

PRL3 induces changes in cellular morphology characteristic of epithelial-mesenchymal transition (EMT), a process important for metastasis 81-85. Cells display an epithelial to fibroblast/mesenchymal-like morphology that is more elongated and spread out, with increased formation of filopodia 11, 41, 69, 86. Furthermore, PRL3 enhances cell adhesion to extracellular matrix (ECM) substrates such as fibronectin and laminin 14, 32, 41, 54, 74, 81. Interestingly, some studies found that co-culture of PRL3 expressing CRC cells with tumor associated macrophages (TAMs) further promoted cell invasion 70, 72, thus emphasizing the importance of crosstalk between PRL3 expressing cells and the surrounding tumor microenvironment.

### Cell cycle deregulation and evasion of apoptosis

Studies have reported arrest at various cell cycle checkpoints - G_1_/S, S, and G_2_/M, upon knockdown or overexpression of PRL3 53, 57, 59, 61, 79, 87, 88. However, others have also found no significant changes in the cell cycle upon PRL3 perturbation 23, 74. These highly varied and conflicting findings are likely due to an endogenous function of PRL3 regulating multiple cell cycle checkpoints. Hence, PRL3 perturbs the cell cycle in a context-dependent manner, given the myriad of genetic mutations in different cell lines that can independently influence the cell cycle.

Though the precise details on how PRL3 influences the cell cycle remains to be fully uncovered, there is consistent evidence that PRL3 has an anti-apoptotic function 22, 34, 47, 53, 57, 59, 61, 67, 79, 87-90. PRL3 protects cells from apoptosis induced by growth factor deprivation stress 67, cytokine deprivation 59, as well as DNA-damaging chemotherapeutic drugs such as doxorubicin, cytarabine 61, and 5-fluorouracil (5-FU) 22, 89. Notably, PRL3 positively regulates the expression of the anti-apoptotic protein, Mcl-1, and loss of PRL3 leads to activation of the intrinsic apoptotic pathway 34, 57, 90.

### Genome instability and mutation

Genome instability has been widely considered a hallmark of cancer that is advantageous for tumor progression, and is characterized by features such as chromosomal instability (CIN), DNA damage and repair defects, as well as loss of telomeric DNA 36. In that regard, PRL3 has been associated with indicators of genome instability in human tumor samples. In PRL3-positive colorectal cancer tissues, markers of CIN were observed 53. A study by Lian *et al* found that overexpression of PRL3 in primary fibroblasts as well as CRC cells leads to abnormalities in telomeric structure and telomere deprotection, which induces a persistent DNA damage response (DDR) that leads to CIN and senescence 91. The study also made a similar observation in transgenic mice, where induction of PRL3 in colon and liver tissues led to telomere deprotection, and this was associated with colon tumorigenesis. In addition, correlation of PRL3 expression with indicators of telomere deprotection and senescence was also seen in tumor samples. The acquisition of further mutations that promote cell cycle checkpoint deregulation can enable senescent cells to achieve replicative immortality, which facilitates cancer progression 36.

In addition, PRL3 also promotes the formation of grossly hyperdiploid and multinucleated cancer cells known as polyploid giant cells (PGCCs) 92. This is facilitated by an increased incidence of incomplete cytokinesis, suppression of DNA damage signaling, which leads to an accumulation of double stranded breaks and genomic instability 92.

### Inducing angiogenesis

Clinically, PRL3 is more significantly expressed in the vasculature of tumor tissue vs normal tissue, such as in the colon 93 and the mammary gland 94. A study of endometrial adenocarcinoma also found a correlation between PRL3 overexpression and Vascular Endothelial Growth Factor (VEGF)-A and VEGF-C expression, as well as greater microvessel density (MVD) and lymphatic vascular density (LVD) 95. In addition, PRL3 expression is associated with vascular invasion in HCC tumors 14, which often display hypervascularity and marked vascular abnormalities 96, 97.

Mechanistically, the earliest evidence that PRL3 was involved in tumor angiogenesis was the observation that PRL3 expressing CHO cells formed micro- and macro-metastatic solid tumors in blood vessels 5. A later study showed that PRL3 expressing cells were able to recruit and enhance vascular formation by human umbilical vein endothelial cells (HUVECs) *in vitro*, as well as recruit host endothelial cells and promote tumor angiogenesis *in vivo* 93. Furthermore, genetic and chemical inhibition of PRL3 abrogates tube formation by HUVEC cultured on Matrigel 98. In addition, inhibition of PRL3 also reduces vascular endothelial permeability of primary human lung microvascular endothelial cells (MVEC) 45. Conversely, overexpression of PRL3 increased migration of cancer cells for metastasis 94. Collectively, these studies point to a pro-angiogenic function of PRL3.

### Deregulating cellular metabolism

Many cancers exhibit the Warburg effect, which is characterized by increased glucose consumption and lactate production 99. PRL3 has been shown to mediate the Warburg effect in CRC cells and multiple myeloma cells 56, 100. In CRC cells, PRL3 promotes increased expression of glycolytic enzymes glucose transporter 1 (GLUT1), hexokinase 2 (HK2), pyruvate kinase M2 (PKM2), and lactate dehydrogenase A (LDHA). Cancer cell proliferation and invasion were abrogated by the lactate dehydrogenase (LDH) inhibitor oxamate, suggesting PRL3-induced metabolic reprogramming contributes to its oncogenic function 100. In MM, cells overexpressing PRL3 were more metabolically active, upregulating both oxidative phosphorylation (OXPHOS) in addition to glycolysis. Enzymes in the serine/glycine pathway were also upregulated, which suggest increased macromolecule biosynthesis 56. Interestingly, the study in MM found these alterations in cancer metabolism were independent of PRL3 phosphatase activity 56.

In addition, a study found that PRL3 knockdown promotes production of mitochondrial superoxide anion with a concomitant increase in mitochondrial membrane potential and induction of cell cycle arrest 101. PRL3 acts as a transcription factor, binding to the promoter of repressor activator protein 1 (RAP1), a protein that regulates the master regulator of mitochondrial biogenesis, peroxisome proliferator-activated receptor gamma coactivator 1 alpha (PGC-1α) 101. Thus, knockdown of PRL3 decreases PGC-1α as well as its downstream genes superoxide dismutase 2 (SOD2) and uncoupling protein 2 (UCP2), both reactive oxygen species (ROS)-detoxifying proteins that prevent the accumulation of ROS. The regulation of a key mediator of mitochondrial function (PGC-1α) by PRL3 suggests that PRL3 could play a critical role in the cellular metabolism. Furthermore, the function of PRL3 as a transcription factor could potentially be a novel mechanism by which PRL3 regulates cellular processes, though this warrants further investigation.

Emerging research has also found PRL3 affects energy metabolism by regulating Mg^2+^ efflux 102-104. The PRL-family (PRL1, PRL2, and PRL3) in general, was shown by both the Tremblay group and Hiroaki group to directly bind and inhibit Mg^2+^ efflux by cyclin M (CNNM) magnesium regulators 105. The interaction between PRL3 and CNNM4 results in decreased Mg^2+^ efflux and increased intracellular Mg^2+^ 104, 105. As intracellular ATP exists in complex with Mg^2+^ and Mg^2+^ is also needed for ATP synthesis, PRL3 overexpressing cells have increased intracellular ATP. Consequently, PRL3 overexpressing cells have a proliferative advantage under glucose-limiting conditions that decrease intracellular ATP levels 104. Interestingly, Hardy *et al* found that Mg^2+^ levels regulate PRL1 and PRL2 mRNA translation through a Mg^2+^-sensitive upstream ORF in the 5-UTR 106. Mg^2+^ depletion and corresponding decrease in intracellular ATP activates the AMPK/mTOR2C pathway, which ultimately leads to increased translation of PRL1 and -2. As this study was done in cell lines that did not express PRL3, it is possible that PRL3 could be regulated by a similar mechanism, but this would need to be confirmed in suitable models expressing PRL3. Nevertheless, the abovementioned findings on PRLs and CNNM/Mg^2+^ regulation open a new area for future studies in defining an exciting mechanism of action of PRL3.

Conversely, others have found that PRL3-overexpressing MM cells were more sensitive to the anti-proliferative effects resulting from glucose uptake blockade by the GLUT1 inhibitor STF-31 56, 107, indicating possible glucose dependency. This finding has potential therapeutic relevance; as GLUT inhibitors have variable efficacy due to metabolic heterogeneity, identifying mutations that predispose tumors to the anti-proliferative and/or cytotoxic effects of glucose uptake blockade are of interest 108. Preliminary evidence demonstrating PRL3 deregulates glucose metabolism and increases sensitivity to GLUT inhibitors suggests it is worth exploring PRL3 expression as a possible biomarker. The interesting insights on the role of PRL3 in deregulating cellular metabolism warrants further study, especially given the seemingly contradictory findings, which are likely because existing studies have been limited to one cancer type (MM). In-depth study into how PRL3 regulates the diverse aspects of cellular metabolism in various cancers would help to explore PRL3 as a biomarker for response to metabolic inhibitors.

### Plasticity and stemness

Recent studies have highlighted a role of PRL3 in cellular plasticity and acquisition of a cancer stem cell (CSC) like state 36, 109, 110. Using a zebrafish model, Johansson *et al* uncovered an endogenous function of PRL3 in preventing differentiation of melanocyte stem cells (MSCs) during regeneration 110. By extension, PRL3 overexpression in melanoma deregulates cellular differentiation to maintain cells in a stem-like state, which is a key feature of cancer progression in general 35. Mechanistically, PRL3 restricts transcription of master transcription factor (MITF)-regulated endolysosomal genes by dephosphorylating and impairing binding of the RNA helicase DDX21. Clinically, dysregulation of endolysosomal pathways has been linked to melanoma progression. Furthermore, the endolysosomal pathway has been implicated in stem cell fate determination as well as cancer stem cells 111, 112.

PRL3 has also been found to promote the transition of ovarian cancer cells to a CSC-like state by increasing the transcription of SRY-Box transcription factor 2 (SOX2), a well-characterized pluripotent factor that maintains cancer stemness 109, 113. PRL3 does so by sequestering histone deacetylase 4 (HDAC4) in the cytoplasm, thereby preventing histone deacetylation and increasing accessibility of the SOX2 promoter to its transcription factor myocyte enhancer factor 2A (MEF2A). Interestingly, this process is independent of PRL3 phosphatase activity, but dependent on the cytoplasmic translocation of PRL3 109.

### Tumor microenvironment

Cancer cells exist in a tumor microenvironment (TME) that has undergone changes to create a niche that is permissive for cancer progression. Of note, increased acidity of the TME drives malignant progression of cancer by promoting ECM degradation and remodeling, cell invasion, as well as creating an immune evasive environment 114, 115. A study by Funato *et al* found that PRL3 stimulates lysosomal exocytosis, which extrudes H+ into the extracellular space. Consequently, PRL3 overexpressing cells become acid addicted, a phenomenon important for PRL3-mediated metastasis 103. A previous study by Machado *et al* had also found a role for increased lysosomal exocytosis in aggressive sarcomas, where the release of soluble hydrolases promotes degradation of the ECM and tumor cell invasion 116.

As previously described, PRL3 is also involved in inhibiting the transcription of endolysosomal genes to promote stemness; this further suggests PRL3 plays a significant role in regulating the endolysosomal system - a hub for multiple biological processes that are perturbed in cancer, such as autophagy, apoptosis, cell adhesion and migration 117.

## Signaling pathways regulated by PRL3

In this section, we highlight how PRL3 is a central node of many oncogenic pathways 118 that are often deregulated in many cancer types, including but not limited to transmembrane growth factor signaling (eg. ErbB), focal adhesion/integrin signaling, cytokine signaling, as well as pathways downstream of p53 and Wnt 71, 86 **(Figure 1)**. This emphasizes PRL3 as a critical player in mediating the hallmarks of cancer and by extension, its relevance as a therapeutic target.

### Receptor Tyrosine Kinase (RTK) signaling

PRL3 has been implicated in RTK signaling, perturbing key downstream pathways commonly deregulated in cancer, such as MAPK/ERK and PI3K/AKT signaling 36. A few examples are highlighted below.

#### Epidermal growth factor receptor (EGFR)

Hyperactivation of EGFR by PRL3 initiates multiple downstream signaling cascades, including MAPK/ERK, JAK/STAT, PI3K/AKT, and p38/JNK. PRL3 does so by transcriptionally downregulating PTP1B, a phosphatase that inhibits EGFR. Consequently, PRL3-overexpressing cells are addicted to EGFR signaling and hypersensitive to EGFR inhibitors, thereby highlighting the potential of PRL3 as a biomarker for response to anti-EGFR therapy such as Cetuximab 48.

##### Platelet-derived growth factor (PDGF)

PDGF signaling is involved in facilitating PRL3 mediated metastasis. PDGF can stimulate PRL3 phosphorylation in a Src-dependent manner 69, a modification that is required for PRL3 mediated cell migration and invasion. In addition, expression of PRL3 induces Src-dependent phosphorylation and activation of PDGFR-β 72, a key player in vascular remodeling and angiogenesis 98.

#### Vascular endothelial growth factor (VEGF)

VEGF, a master regulator of tumor angiogenesis 119, 120, has been implicated in PRL3-induced angiogenesis 24, 95, 98. Clinically, PRL3 overexpression is associated with VEGF expression, tumor angiogenesis and lymph node metastasis 24, 95. VEGF induces transcription of PRL3, which is needed for tube formation by HUVEC cultured on Matrigel 98. Conversely, inhibiting PRL3 downregulated VEGF by downregulating ERK1/2 signaling through reduction of phospho-ERK1/2 24, 95.

### Focal adhesion signaling

There is significant evidence that PRL3-mediated migration, invasion, and metastasis is regulated by integrin/Rho signaling, with the involvement of kinases like Src and focal adhesion kinase (FAK), as well as various matrix metalloproteinases (MMPs).

PRL3 was shown to be co-amplified and co-expressed with FAK in HCC. The involvement of PRL3 results in the phosphorylation of FAK which then activates the p38 and PI3K/AKT pathway, mediating the oncogenic effect of PRL3 in HCC cells 14.

In addition, PRL3-induced cell migration is Src-dependent and can be abolished using a Src inhibitor 81, 121. Mechanistically, PRL3 downregulates C-terminal Src kinase (Csk), a negative regulator of Src, to induce Src activation 81. This leads to increased activity of p130^Cas^ - a scaffold protein which localizes to focal adhesion complexes to coordinate downstream pathways mediating cell migration, as well as ERK1/2, signal transducer and activator of transcription 3 (STAT3), which promote cell proliferation and invasion 81, 122, 123. Conversely, PRL3 can be phosphorylated and activated by Src, triggering downstream signaling mediated by Rho GTPases to promote cell migration and invasion 69, 124. Interestingly, a study found PRL3 dephosphorylates and inactivates vav guanine nucleotide exchange factor 1 (VAV1), a regulator of Rho GTPases that is critical for T cell receptor (TCR) signaling. PRL3 is coamplified with the known oncogenic driver MYC, and they synergize to sustain T-ALL tumor growth. The re-activation of TCR signaling upon PRL3 inhibition kills T-cell acute lymphoblastic leukemia (T-ALL) cells 63.

Furthermore, there is a significant correlation of PRL3 with the expression of various MMPs, such as MMP2 and MMP9 in HCC 13, 125. These zinc-dependent endoproteinases break down ECM of surrounding tissues to facilitate the invasion of tumor cells and metastasis 126, 127. Mechanistically, studies in CRC cells found that PRL3-mediated invasion and migration was dependent on MMP7 128, and PRL3 also enhanced the gelatinolytic function of MMP2 122.

### Cytokine signaling

PRL3 has also been implicated in cytokine signaling, such as pathways downstream of pro-inflammatory cytokines interleukin-6 (IL-6) and tumor necrosis factor alpha (TNFα), with the involvement of key transcription factors regulating the inflammatory response, nuclear factor kappa-light-chain-enhancer of activated B cells (NF-κB) and STAT3 50, 66, 70-72, 83, 85, 129.

STAT3 is a direct transcriptional regulator of PRL3 and mediates IL-6-dependent increase in PRL3. Conversely, inhibition of PRL3 reduces nuclear accumulation of STAT3, thereby downregulating downstream targets c-myc, cyclin D1, and the anti-apoptotic proteins Mcl-1, Bcl-xL and Bcl-2. This leads to decreased cell migration and survival in both *in vitro* and *in vivo* systems 57, 130.

Ectopic expression of PRL3 in CRC cells increases secretion of the cytokine TNFα, which activates NF-κB in an autocrine manner, leading to cell growth via increased G_2_/M transition 50, induction of EMT 83, as well as increased secretion of angiogenesis associated proteins such as VEGF-A 85. This is achieved in part by NF-κB-dependent upregulation of potassium calcium-activated channel subfamily N member 4 (KCNN4), a Ca^2+-^activated K^+^ channel which has been shown to promote cell proliferation, invasion, and metastasis by activating MAPK/ERK and PI3K/AKT signaling 131. Furthermore, TNFα secreted by PRL3 overexpressing cells can stimulate surrounding TAMs to produce proinflammatory cytokines IL-6 and IL-8, creating a paracrine loop that further induces EMT 85 and promotes metastasis 70. PRL3 overexpressing cells also produce more chemokine ligand 26 (CCL26), which engages the chemokine receptor type 3 (CCR3) on TAMs, inducing TAM infiltration *in vivo* to promote tumor growth 72, 85. In addition, NF-κB transcriptionally regulates PRL3 expression downstream of TNFα. Knockdown of PRL3 induces G_1_ cell cycle arrest and senescence in triple-negative breast cancer (TNBC) cells, and continued suppression leads to increased secretion of TNFα, which activates the extrinsic apoptotic pathway in an NF-κB dependent manner 54.

Evidently, the reciprocal regulation between PRL3 and TNFα/IL-6 illustrates the important role of cytokine signaling in PRL3 expressing tumors as well as their surrounding microenvironment.

### p53 signaling

Mutual regulation exists between PRL3 and the tumor suppressor p53. PRL3 is known to be a direct transcriptional target of p53, whereas PRL3 negatively regulates p53 protein levels 89, 132, 133. In non-transformed primary cells, both knockdown and overexpression of PRL3 levels triggers G_1_ arrest, alluding to a requirement for basal levels of PRL3 for proper cell cycle progression 132. In cancer cells - which often deregulate p53, PRL3 downregulates p53, which can lead to increased cell proliferation and clonogenicity 133, increased G_1_-S cell cycle progression 61, as well as inhibition of apoptosis 89. In general, PRL3 promotes proteasomal degradation of p53 protein through increased activity of mouse double minute 2 homolog (Mdm2), the negative regulator of p53 89. Several mechanisms have been described; in breast cancer cells, the Mdm2 inhibitor p14^ARF^ is downregulated 133, and activated Akt phosphorylates and stabilizes Mdm2 in colorectal HCT116 p53 (+/+) cells 89. Upregulation of PIRH2, another p53-induced ubiquitin-protein ligase which promotes its degradation, was also observed 89. Thus, we can see that deregulation of PRL3 and p53 in cancer cells interact to disrupt homeostatic control of the cell cycle and cell proliferation.

### PTEN/PI3K/AKT/mTOR signaling

PRL3 activates the PI3K/AKT pathway by downregulating its key negative regulator PTEN, leading to increased cell proliferation, EMT, migration, and invasion 15, 82. In the context of melanoma progression, PRL3 dephosphorylates Na^+^/H^+^ exchanger regulating factor 1 (NHERF1), an interactor of PTEN, promoting the nuclear to cytoplasmic translocation of both NHERF1 and PTEN. The differential localization of PTEN leads to phosphorylation and activation of AKT 134. In addition, AKT activation was also observed upon knockdown of polyC-RNA-binding protein 1 (PCBP1), which inhibits translation of PRL3 mRNA by preventing its incorporation into polyribosomes. In HCC tumors, a negative regulator of PCBP1, the long non-coding RNA (lncRNA) PCBP1-AS1, is highly expressed compared to adjacent normal tissue, and correlates with increased metastasis and poorer patient survival. The increase in cell proliferation and metastasis induced by PCBP1-AS1 overexpression was shown to be dependent on the PCBP1-PRL3-AKT axis, which consequently promotes tumor growth and metastasis *in vivo* 7, 135.

Activation of mechanistic target of rapamycin complex 1 (mTORC1) signaling by PRL3 drives motility, invasion, as well as tumorigenesis 104, 136. PRL3 increases binding of mTOR to RagB/C GTPases, which facilitates mTORC1 recruitment to lysosomal membranes 136. In turn, RagB/C inhibit both autophagic and proteasomal degradation of PRL3 137. Furthermore, PRL3 promotes canonical autophagic flux in addition to serving as an autophagic substrate, thereby creating a negative feedback loop 44. Consequently, PRL3-driven autophagy is associated with AKT activation and increased cell growth 44.

### Wnt signaling

Research has shown PRL3 mediates its oncogenic function in AML by activating canonical Wnt signaling 60, 138, Consequently, AML cells overexpressing PRL3 have enhanced sensitivity towards β-catenin inhibition both *in vitro* and *in vivo* 138. In addition, combinatorial inhibition of PI3K/AKT/mTOR and Wnt/β-catenin signaling is synthetic lethal specifically in cells with high PRL3 levels 139. Thus, Wnt/β-catenin signaling, in conjunction with other signaling pathways, plays a significant role in mediating downstream effects of PRL3.

## PRL3-targeted therapy

The tumor-specific expression of PRL3 across a broad range of cancer types makes it an attractive therapeutic target. Significant work has been done in developing PRL3-targeted therapy, which includes the identification and optimization of small molecule inhibitors against PRL3 22, 47, 49, 68, 140-149, as well as the development of antibody-based therapy 9, 150-153. We will briefly discuss the usage of PRL3 inhibitors, which has been reviewed in detail by Wei *et al* 154, and highlight recent advances in anti-PRL3 immunotherapy, where a first-in-class humanized monoclonal antibody (mAb) known as PRL3-zumab is currently in Phase II clinical trials (ClinicalTrials.gov identifier: NCT04118114; NCT04452955).

### Small molecule inhibitors against intracellular PRL3

Much work on PRL3 inhibitors has focused on computationally screening small molecule libraries to identify potential lead compounds for further *in vitro* validation and optimization 45, 49, 77, 141, 144-149, 155. Rhodanine-based compounds have been of key interest after this chemotype was shown to have inhibitory activity against PRL3 through a high throughput screening of a chemical library in 2006 141. Rhodanine derivatives have been demonstrated to inhibit PRL3-mediated cell proliferation, migration, invasion, as well as induce cell cycle arrest and apoptosis in cancer cells 22, 47, 68, 143. Most notably, the compound BR-1 was shown to reduce tumor growth *in vivo* and synergize *in vitro* with the multikinase inhibitor Sorafenib 143. Despite these promising results, an overwhelming majority of studies on small molecule inhibitors against PRL3 have only been done in cell line models, and further work *in vivo* needs to be done before PRL3 inhibitors can move into clinical trials. This involves overcoming the challenge of limited selectivity in the design of PRL3 inhibitors; the high degree of similarity between PRL3 and its other family members PRL1 and PRL2 makes it challenging to develop an inhibitor specific to PRL3 147, 156. Furthermore, PRL3 inhibitors could also target other structurally similar phosphatases like the tumor suppressor PTEN 156. Hence, there is a need to tap on existing knowledge around PRL3 structural biology to address the issue of off-target effects.

### Antibody-based therapy against intracellular PRL3

A major challenge in cancer therapy is a lack of target specificity for cancer cells, resulting in damage to normal tissues. Antibody-based therapy have greater specificity over standard chemotherapy regimens and thus improved efficacy. Hence, the research efforts of the Zeng group have focused on using PRL3 antibodies to target PRL3 expressing tumors, as opposed to small inhibitors. Extensive work has led to the development of a first-in-class humanized monoclonal antibody (PRL3-zumab) against PRL3, a novel cancer immunotherapy against an intracellular antigen 153. Using orthotopic cancer mouse models, it was shown that PRL3-zumab reduces tumor growth, inhibits metastatic tumors, reduces tumor recurrence, and increases survival 8, 150, 151, 153. In addition, PRL3-zumab can serve as a form of adjuvant immunotherapy to eliminate stem-cell like PGCCs that remain after tumor removal, thereby preventing relapse 92. The abovementioned effects are seen in tumors that express PRL3 but not in PRL3-negative tumors, and PRL3-zumab also showed no cross-reactivity with the closely related homologs PRL1 and PRL2. Hence, this confirms the specific binding of PRL3-zumab to its target antigen, PRL3 8, 9, 150, 151, 153. The substantial pre-clinical data paved the way for PRL3-zumab to enter clinical trials, and the immunotherapeutic is currently in Phase II clinical trials in Singapore (ClinicalTrials.gov identifier: NCT04118114), USA (ClinicalTrials.gov identifier: NCT04452955) and China (China Drugtrials.org.cn identifier: CTR20211180), with the Phase II clinical trial in the US almost completed. In both the Phase I and Phase II trials, PRL3-zumab has shown a strong safety profile with no serious adverse events (SAEs), infusion reactions, dose-limiting toxicities (DLTs), or drug-related deaths.

PRL3-zumab targets PRL3 intracellular oncoprotein, which begets the question of the antibody's mechanism of action (MOA), given the general assumption that antibodies can only target antigens located on the cell surface as they are too large to enter the cell 157.

However, several lines of evidence have challenged this assumption 158, 159. In a major proof-of-concept study, Guo *et al* generated three mAbs against their respective intracellular proteins - PRL3, EGFP, and the polyomavirus middle T oncoprotein (mT) 160, 161. The study found the mAbs induced tumor regression in immunocompetent wild type C57BL/6 mice specifically expressing the target protein (PRL3 or EGFP), as well as in MMTV-PyMT transgenic spontaneous breast cancer models specifically expressing the target intracellular mT. For example, anti-PRL3 mAb induced tumor regression of PRL3-positive tumors but not PRL3-negative tumors, thereby highlighting the specificity of mAbs for their respective targets. Furthermore, proteins that are located intracellularly in normal cells have been shown to be externalized on the surface of tumor cells. These include viable therapeutic targets such as heat-shock protein 70 (HSP70), heat-shock protein 90 (HSP90), glucose-regulated protein 78 and 94 (GRP78 and GRP94), vimentin, estrogen receptor-alpha variant 36 (ER-α36), and feto-acinar pancreatic protein (FAPP) 162. In the pre-clinical setting, mAbs designed specifically against known externalized epitopes have been developed by GRP94 163, HSP70 164. In addition, early studies have shown that tumor cell membranes are generally more permeable than normal cells 165, which is favorable for secretion and externalization of intracellular antigens.

In the case of PRL3, Min *et al* found evidence that the intracellular PRL3 oncoprotein is externalized on the cell surface in tumor cells *in vivo* and serum-starved cells *in vitro*; this phenomenon is not observed in normal cultured cells *in vitro*, suggesting that stresses in the tumor microenvironment promote externalization of PRL3, and this can be recapitulated *in vitro* by subjecting cells to serum starvation 8. PRL3 mini-body (lacking Fc domain) showed no therapeutic effect, suggesting PRL3-zumab recruits immune cells such as B cells, natural killer (NK) cells, and macrophages in an Fc receptor (FcR)-dependent manner, which implies the involvement of antibody-dependent cellular cytotoxicity (ADCC) and antibody-dependent cellular phagocytosis (ADCP) in the elimination of cancer cells 8. Notably, studies utilizing more than 4000 mice from 8 different strains demonstrated the therapeutic efficacy of anti-PRL3 mAb against PRL3+ tumors could be T-cell independent in nude mice models 166. In addition, PRL3 is secreted extracellularly on the surface of exosomes, a possible mechanism behind the 'inside-out' phenomenon of PRL3, which also serves as bait for PRL3-zumab. It has been hypothesized by Ferrone *et al* 161 that resultant antigen-antibody complexes can be processed by dendritic cells for antigen presentation and subsequent T cell and NK cell activation, though there has thus far been found no evidence of T cell involvement. 8, 153, 159. As PRL3 can be found on the cell membrane and endosome (where exosomes can originate) when prenylated 3, it is plausible that PRL3-zumab mediates its effects by targeting PRL3 antigen externalized on the cell membrane and exosomes.

## Perspectives

### PRL3 - an attractive therapeutic target for a broad range of cancer types and beyond

There is compelling evidence that PRL3 is an attractive therapeutic target for cancer. PRL3 is a tumor-specific antigen overexpressed across a broad range of tumor types and largely absent in matched normal tissue. PRL3 protein perturbs a myriad of cellular functions and signaling pathways commonly deregulated in cancer, establishing it as an oncogenic driver with a key role in cancer progression.

Recent findings have uncovered previously undescribed functions of PRL3, such as its endogenous function in development, and its ability to deregulate cellular metabolism and alter the TME. Aside from advancing basic knowledge on PRL3, understanding the signaling pathways perturbed by PRL3 can help expand its potential as a biomarker. For example, the association of PRL3 expression with addiction to EGFR signaling highlights the potential of PRL3 to serve as a biomarker for response to anti-EGFR therapy. Work demonstrating PRL3-induced activation of mTORC1 signaling as well as incipient evidence that PRL3 mediates the Warburg effect and glucose dependency suggests PRL3 could contribute to the growing need to identify genetic mutations associated with metabolic vulnerabilities that predict sensitivity to inhibitors targeting cancer metabolism 167.

In addition, several recent studies have uncovered phosphatase-independent functions of PRL3, which has a greater diversity of binding partners apart from enzymatic substrates. Some have even proposed that PRL3 phosphatase activity is dispensable for its oncogenic function 76, 109, 168. For example, ablating CNNM binding function of PRL3 alone prevented metastasis of B16 mouse melanoma cells, whereas a phosphatase-dead PRL3 mutant that could still bind CNNM showed no significant change in tumorigenesis 104. The suggestion that PRL3 phosphatase activity is dispensable for its oncogenic function is provocative, given the overwhelming amount of contradictory evidence. However, Kozlov *et al* pointed out many studies utilized PRL3 mutants deficient in both CNNM binding and phosphatase activity, making it difficult to dissect the contribution of each individual function. By leveraging the wealth of information on PRL3 structure 157-159, future studies utilizing PRL-3 mutants that perturb specific domains could help better address this question 169-171. Importantly, the discovery of these novel phosphatase-independent functions of PRL3 suggests that PRL3 could regulate a broader range of biological processes that have yet to be uncovered 76, 103, 109. In particular, the role of PRL3 in regulating magnesium transport is an emerging area of interest, with several studies demonstrating it is a mechanism by which PRL3 alters cellular metabolism to promote cell proliferation and tumorigenesis. Overall, further investigation would help identify contexts in which PRL3 could influence response to therapy targeting a particular deregulated biological process.

By identifying diseases in which PRL3-regulated pathways are perturbed, it is tempting to speculate we could apply PRL3-targeted therapy to other relevant diseases. For example, PRL3 directly binds to CNNM magnesium regulators, whose suppression has been implicated not only in cancer, but also in other diseases such as hypertension and schizophrenia. In fact, preliminary efforts have been made to indirectly enhance CNNM expression via treatment with PRL3 inhibitors as a therapeutic strategy 102-104. Thus, it would be worth exploring the therapeutic potential of targeting PRL3 in the context of other diseases, thereby broadening the scope of its application in the clinic.

### PRL3-zumab - a different take on immunotherapy

**Figure 2:**
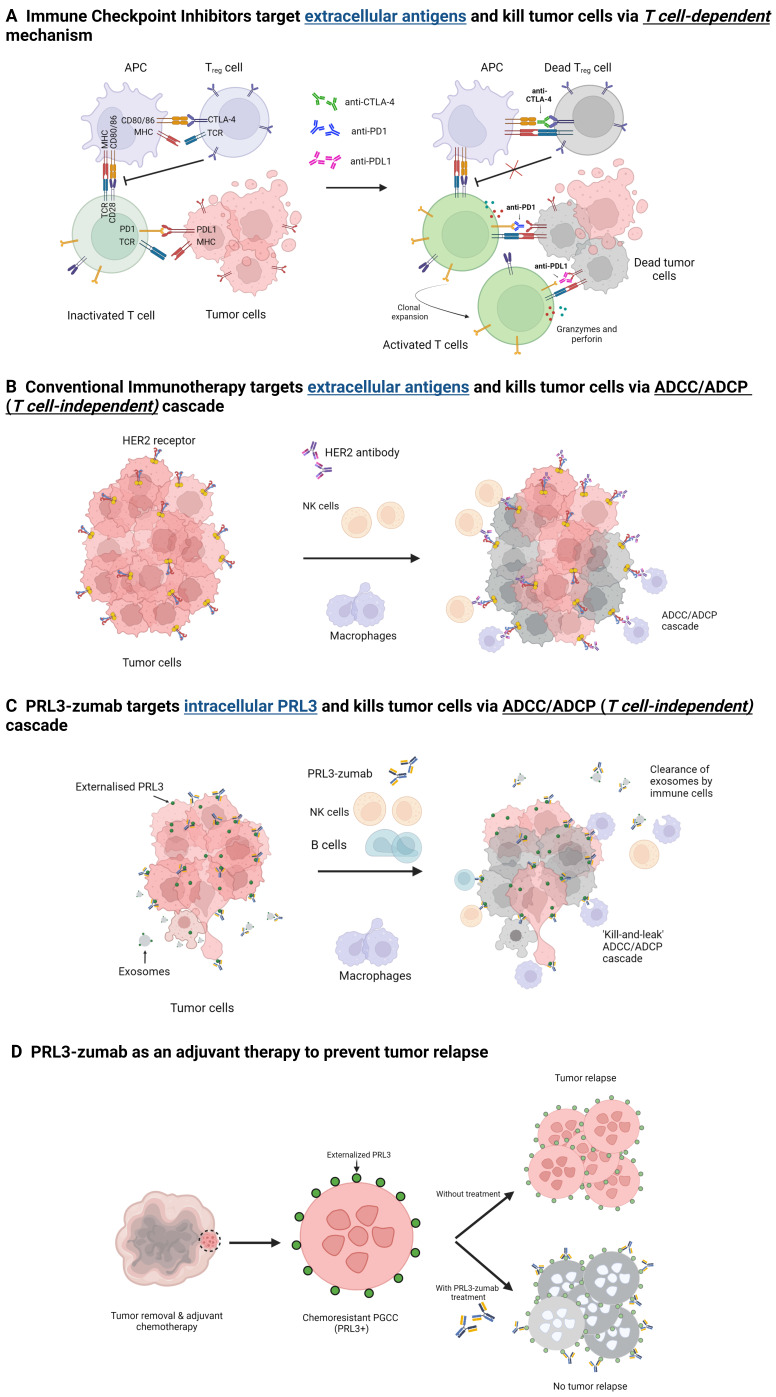
Schematic illustrations of the MOAs of immune checkpoint inhibitors, conventional immunotherapy, and PRL3-zumab. **A**) Immune checkpoints to prevent T cell activation (left). Immune checkpoint inhibitors block CTLA-4, PD-1 and PD-L1 and this blockade allows the binding of MHC with TCR in T_reg_ and T cells, leading to the clonal expansion and activation of T cells. Activated T cells release granzymes and perforin which consequently kill the tumor cells (right). **B**) Tumor cells with extracellular targets (left). Binding of antibody to extracellular target triggers the antibody-dependent cellular cytotoxicity and phagocytosis (ADCC/ADCP) cascade, resulting in the killing of tumor cells by immune cells. **C**) Tumor cells with externalized intracellular PRL3 (left). Binding of PRL3-zumab to the externalized intracellular PRL3 in TME leads to ADCC/ADCP cascade and killing of the tumor cells akin to conventional immunotherapy. PRL3-zumab can also bind to PRL3 on the surface of secreted exosomes for clearance of exosomes to prevent metastasis. **D**) Chemo-resistant PRL3+ polyploid giant cancer cells (PGCCs) have stem-cell like properties, which remain after tumor removal and adjuvant chemotherapy, can initiate tumor relapse. PRL3-zumab could serve as an adjuvant therapy to eliminate PGCCs and prevent tumor relapse. Created with BioRender.com.

PRL3-zumab, the first-in-class humanized antibody, is currently in Phase II clinical trials in Singapore, USA, China, and Malaysia. PRL3-zumab is a novel cancer immunotherapy at the forefront of PRL3-targeted therapy (**Figure 2**). As a therapeutic mAb, PRL3-zumab has demonstrated a strong safety profile in Phase I and Phase II clinical trials. In general, therapeutic mAbs avoid the toxic side effects associated with conventional chemotherapy. Furthermore, targeted therapy is more specific than small molecule inhibitors, thereby avoiding toxic side effects 172. However, the safety profile and effectiveness of therapeutic mAbs are dependent on the suitability of the targeted antigen, i.e. its specificity and prevalence in tumor cells. A tumor-specific target antigen is critical to prevent the drug from attacking normal tissues, thereby reducing adverse side effects in patients.

The ubiquitous and tumor specific expression implies that targeting PRL3 would be appropriate in multiple cancer types with few side effects. PRL3 plays an essential role not just in the biology of tumor cells, but also the tumor microenvironment. Hypothetically, targeting PRL3 would yield a two-pronged approach of attacking tumor cells as well as the TME, thereby shrinking the tumor as well as creating an unfavorable niche for cells to proliferate. Furthermore, PRL3-zumab can act as adjuvant therapy to eliminate residual tumor cells refractory to other forms of treatment. Targeting PRL3 would thus be a unique therapeutic strategy for eradicating a tumor in its entirety.

Notably, PRL3 is an intracellular oncoprotein, which challenges the widespread assumption that antibodies can only target extracellular proteins 159, 166. Because of this assumption, the search for tumor associated antigens have focused on surface proteins which include cell surface differentiation antigens, growth factors, as well as vascular targets involved in angiogenesis 173. PRL3-zumab is a proof of concept that intracellular oncoproteins are viable targets for therapeutic mAbs. Hence, by expanding the repertoire of potential targets to include intracellular tumor antigens, we can further the use of therapeutic mAbs in cancer immunotherapy.

In recent years, much focus has been on developing immune checkpoint inhibitors (ICIs), monoclonal antibodies that target negative regulators of T cell activation, namely CTLA-4 and PD-1 on T_reg_ cells or T cells and PD-L1 on tumor cells 173-176. Antibodies against CTLA-4, PD-1 and PD-L1 can block the binding of CTLA-4 & CD80/86 on antigen presenting cells (APC) or the binding of PD-1 and PD-L1 on tumor cells, which can lead to binding of TCR and MHC causing T cell activation. Activated T cells release granzymes and perforin which ultimately results in tumor cell killing and decreased metastasis 173-177. The use of ICIs such as anti-CTLA-4 and notably anti-PD-1/PD-L1 - which has been shown to have a better safety profile - has contributed significantly to the improvement of patient outcome 175-178. However, there are two key issues/limitations that need to be addressed.

Firstly, ICIs can result in immune-related adverse events (irAEs) due to activated T cell populations that can infiltrate a broad spectrum of organs. The most fatal irAEs of major concern include encephalitis, hepatitis, myocarditis, and pneumonitis. As these side effects can affect many different organs, and also differ from those associated with conventional chemotherapy, clinicians face the challenge of learning how to diagnose and manage irAEs with the growing use of ICIs 177, 179.

Secondly, there is a heterogeneous benefit rate for ICIs. The benefit rate is 10-20% in solid tumors, 40-50% in melanoma and non-small cell lung cancers (NSCLC), and 60-75% in Hodgkin lymphoma 180.

Multiple factors influence response to ICIs, include degree of tumor infiltrating T cells, target expression, as well as tumor mutational burden (TMB). In addition, different patients also have variable risk for irAEs. Hence, much research has focused on identifying biomarkers to predict patients that will benefit from ICIs 178, 181-183.

To this end, we propose that PRL3-zumab, which targets a tumor specific antigen widely expressed in various tumor types, can address these issues through its excellent safety profile, providing an alternative for patients that do not respond well to ICIs. PRL3-zumab could also benefit a broad set of patients given its expression in a wide range of tumor types. The MOA of PRL3-zumab involves NK cells, macrophages, and B cells 159, in contrast to ICIs, which rely on T-cell activity. Diversifying strategies by targeting both humoral and cell immunity provides a more comprehensive approach to immunotherapy and would ultimately translate to more treatment options for patients, thereby increasing the chances of finding a suitable therapy to improve clinical outcome. With further clinical studies, PRL3-zumab could potentially serve as an alternative immunotherapy option for patients, including those who are resistant and/or experience adverse side effects to ICIs.

## Conclusions

PRL3 has been established as a key oncogenic driver that promotes cancer progression and metastasis. PRL3 overexpression in multiple cancers has pleiotropic effects, causing cells to acquire hallmark traits such as sustained proliferative signaling, replicative immortality, genome instability and mutation, resistance to cell death, angiogenesis etc. Recent findings have also expanded our knowledge of PRL3-mediated functions, including its endogenous function in regulating stem cell differentiation during development, and its influence on the TME. Importantly, its tumor specific expression in a broad range of cancers and absence in normal tissues make it an attractive therapeutic target. PRL3-zumab, a first-in-class humanized monoclonal antibody targeting PRL3, has shown promising results, including an excellent safety profile, in Phase I and II clinical trials. This novel immunotherapeutic against an intracellular target challenges a fundamental assumption that only cell surface proteins are candidates for monoclonal antibody therapy, alluding to the untapped potential of other tumor-specific antigens that have yet to be discovered. Importantly, PRL3-zumab could potentially serve as an alternative immunotherapy option for patients who are unsuitable for treatment with ICIs, thereby addressing an urgent unmet medical need.

## Figures and Tables

**Table 1:** PRL3 expression in primary and metastatic tumors

Year of first report	Type of cancer	Expression			
		*Primary*		*Metastatic*	
		% of tumors	*Method*	% of tumors	*Method*
**2001** [Saha S. *et al*]	Colorectal	**44.6** 11	ISH	Lymph nodes: **100**; Liver: **91** Lung: **86**; Brain: **100**; Ovary: **100** 12	ISH
**2004** [Wu X. *et al*]	Liver	*Hepatocellular carcinoma (HCC)* **p = <0.001 in T vs N** 13, 14 **p = <0.01 in T vs N** 15	RT 13, 15 IHC 14		
		*Intrahepatic cholangiocarcinoma (ICC)* **47.1** 16	IHC	Lymph nodes: **80.6** 16	IHC
**2004** [Miskad UA. *et al*]	Gastric	**70.4** 17, 18	IHC	Lymph nodes: **74.1** 18	ISH
**2004** [Parker BS. *et al*]	Breast	**70.7** 19	IHC	Positively correlated with lymph node metastases 19	
**2005** [Polato F. *et al*]	Ovarian	Stage III>Stage I 20	RT		
**2007** [Kong L. *et al*]	Brain	Grade IV: **50** 21	IHC		
**2008** [Liu YQ. *et al*]	Esophagus	**78** 22	IHC	**96** 22	IHC
**2008** [Fagerli UM. *et al*]	Myeloma	**90** 23	IHC		
**2009** [Ming J. *et al*]	Lung	Stage III: **79.2** 24	IHC	Lymph nodes: **81.5** 24	IHC
**2011** [Ma Y. *et al*]	Cervix	**100** 25	IHC	Lymph nodes: **100** 25	IHC
**2011** [Laurent C. *et al*]	Eye			High in metastases 26	FC
**2011** [Hassan NM. *et al*]	Oral	Stage III/IV> Stage I/II 27	IHC		
**2012** [Zhou J. *et al*]	Myeloid Leukemia	**47** 28	IHC		
**2013** [Guzińska-Ustymowicz K. *et al*]	Endometroid	**100** 29	IHC	Lymph nodes: **100** 29	IHC
**2015** [Fang XY. *et al*]	Skin	48.6% of patients - high expression correlates with melanoma-specific death 30	GE		
**2016** [Vandsemb EN. *et al*]	Prostate	**100** 31	IHC	Lymph nodes: **100** 31	IHC
**2017** [Hjort MA. *et al*]	Lymphoblastic Leukemia	Higher in ALL cells than normal cells 32	RT, FC		
**2017** [Sun F. *et al*]	Kidney [Wilms']	**19** 33	IHC		
**2018** [Hjort MA. *et al*]	Hodgkin Lymphoma	**16** 34	IHC		

RT: RT-PCR; ISH:* In situ* hybridization; GE: Gene expression (Bioinformatics); IHC: Immunohistochemistry; FC: Flow cytometry

**Table 2:** Comparison between IHC and WB in the detection of PRL3 protein expression in tumor samples

Type of cancer	IHC		WB	
	n	PRL3+ [%]	n	PRL3+ [%]
Liver	14	14.3	20	80
Lung	155	29	10	90
Colorectal	301	20.6	10	70
Breast	173	12.7	10	90
Stomach	18	16.7	14	86
Kidney	12	8.3	18	72
Prostate	53	9.4	4	100
Pancreas	126	33.3	7	86
Bladder	14	21.4	34	71
Brain	20	10		
Esophagus	18	27.8		
Cervix	15	20		
Squamous	66	37.7		
Ovary	12	8.3		
Skin	8	37.5		
Total/Average [%]	1008	22.3	151	80.6

IHC: Immunohistochemistry; WB: Western blotting
